# Comparison of 21 artificial intelligence algorithms in automated diabetic retinopathy screening using handheld fundus camera

**DOI:** 10.1080/07853890.2024.2352018

**Published:** 2024-05-13

**Authors:** Anna-Maria Kubin, Petri Huhtinen, Pasi Ohtonen, Antti Keskitalo, Joonas Wirkkala, Nina Hautala

**Affiliations:** a Department of Ophthalmology, Oulu University Hospital, Oulu, Finland; b Research Unit of Clinical Medicine, Oulu, Finland; c Medical Research Center, University of Oulu, Oulu, Finland; d Optomed, Oulu, Finland; e Research Service Unit, Oulu, Finland; f The Research Unit of Surgery, Anesthesia and Intensive Care, Oulu University Hospital and University of Oulu, Oulu, Finland

**Keywords:** Diabetes, diabetic retinopathy, screening, artificial intelligence, handheld fundus camera

## Abstract

**Background:**

Diabetic retinopathy (DR) is a common complication of diabetes and may lead to irreversible visual loss. Efficient screening and improved treatment of both diabetes and DR have amended visual prognosis for DR. The number of patients with diabetes is increasing and telemedicine, mobile handheld devices and automated solutions may alleviate the burden for healthcare. We compared the performance of 21 artificial intelligence (AI) algorithms for referable DR screening in datasets taken by handheld Optomed Aurora fundus camera in a real-world setting.

**Patients and methods:**

Prospective study of 156 patients (312 eyes) attending DR screening and follow-up. Both papilla- and macula-centred 50° fundus images were taken from each eye. DR was graded by experienced ophthalmologists and 21 AI algorithms.

**Results:**

Most eyes, 183 out of 312 (58.7%), had no DR and mild NPDR was noted in 21 (6.7%) of the eyes. Moderate NPDR was detected in 66 (21.2%) of the eyes, severe NPDR in 1 (0.3%), and PDR in 41 (13.1%) composing a group of 34.6% of eyes with referable DR. The AI algorithms achieved a mean agreement of 79.4% for referable DR, but the results varied from 49.4% to 92.3%. The mean sensitivity for referable DR was 77.5% (95% CI 69.1–85.8) and specificity 80.6% (95% CI 72.1–89.2). The rate for images ungradable by AI varied from 0% to 28.2% (mean 1.9%). Nineteen out of 21 (90.5%) AI algorithms resulted in grading for DR at least in 98% of the images.

**Conclusions:**

Fundus images captured with Optomed Aurora were suitable for DR screening. The performance of the AI algorithms varied considerably emphasizing the need for external validation of screening algorithms in real-world settings before their clinical application.

## KEY MESSAGES

**What is already known on this topic?** Diabetic retinopathy (DR) is a common complication of diabetes. Efficient screening and timely treatment are important to avoid the development of sight-threatening DR. The increasing number of patients with diabetes and DR poses a challenge for healthcare.

**What this study adds?** Telemedicine, mobile handheld devices and artificial intelligence (AI)-based automated algorithms are likely to alleviate the burden by improving efficacy of DR screening programs. Reliable algorithms of high quality exist despite the variability between the solutions.

**How this study might affect research, practice or policy?** AI algorithms improve the efficacy of screening and might be implemented to clinical use after thorough validation in a real-life setting.

## Introduction

Future projections estimate that 643 million people will have diabetes by 2030 and 783 million by 2045 [1]. Diabetes is associated with several complications, which may lead to significant morbidity posing a challenge for healthcare providers. Diabetic retinopathy (DR) is one of the major complications of diabetes, estimated to be the leading cause of blindness among working-age adults globally [2,3]. Prevalence of DR varies from 37% up to 94–97% in patients with long-term duration of type 1 and from 20% to 40% in those with type 2 diabetes [2,3]. Among individuals with diabetes, approximately 6% and 4% develop sight-threatening DR or clinically significant macular oedema, respectively [3]. Regular screening for DR mostly by fundus photography is an efficient way to avoid the development of severe DR and irreversible loss of vision [4,5].

Strong evidence of the importance and cost-effectiveness of DR screening has been addressed [4,6–8]. Implementation of DR screening programs varies greatly throughout the world, and successfully established screening protocols with high coverage exist on national level only in a limited number of countries. In Finland, for example, screening for DR is well-organized according to national screening guidelines and utilizing telemedicine especially in the rural areas of the country [9,10]. Along with optimized diabetes care and timely treatment of DR, this has substantially reduced the risk of visual loss [4,5]. The increasing prevalence of diabetes is likely to increase the number of patients who benefit from regular access to DR screening. However, resources for nationwide screening programs are scarce in many countries. In rural areas and low income countries, the need to travel vast distances and the lack of retinal cameras, trained healthcare professionals and ophthalmologists are important barriers to the clinical implementation of DR screening [11,12]. Current screening systems also rely greatly on human graders, a resource both costly and in limited supply. Implementation of telemedicine solutions, mobile handheld devices and artificial intelligence (AI)-based automated analysis for DR might help to solve these challenges by alleviating the burden for screening and improving cost-effectiveness [13–16].

Recent studies have shown indisputable benefits of AI-solutions based on deep learning technology for DR grading [8,11–13,17]. However, the outcomes from different algorithms are notably varying and the comprehensive real-world testing is limited. The aim of the current study is to compare the performance and suitability of 21 existing AI-based algorithms on screening of referable DR in a real-world setting. A mobile handheld fundus camera was used to gather the real-world clinical data.

## Patients and methods

This study was carried out at Oulu University Hospital. The study followed the tenets of the Declaration of Helsinki, and it was conducted with the approval of the Oulu University Hospital Research Committee (175/2016). Informed written consent was obtained from all participants. Complete anonymity was adhered to, and the article does not include any data that may identify the person.

A total of 156 patients with either type 1 or type 2 diabetes were included. The colour and red-free papilla- and macula-centred fundus images were taken from both eyes of each patient with the handheld Optomed Aurora fundus camera with a 50° field of view, non-mydriatic operation, nine internal fixation targets and WLAN connectivity for transmitting images to the PC (Optomed Aurora, Optomed, Oulu, Finland). A total of 1248 images (eight images per patient) were analysed by the retina specialists and 624 colour images were analysed by each of the algorithms. The first 106 consecutive patients included in the study were attending screening of DR in the mobile unit EyeMo utilizing telemedicine-based technologies. To include more severe cases of DR and other retinal changes (age-related macular degeneration, retinal vein occlusion, etc.) in the study, further 50 patients were evaluated in the hospital’s outpatient eye clinic. Demographics of the participants were not collected. Fundus images were analysed by using high-quality 27″ screens. The images were manually graded by two retina specialists using the five-scale grading system developed by the Finnish Current Care Guidelines [10]. The stages 0 (no DR) and 1 (mild nonproliferative diabetic retinopathy (NPDR)) were considered as non-referable DR, and stages 2 (moderate NPDR), 3 (severe NPDR) and 4 (proliferative diabetic retinopathy (PDR)) as referable in the DR screening program. The stage of DR and the need for a referral to an ophthalmologist were determined according to an eye with more severe DR. Other retinal abnormalities were also documented for attention. The human graders were allowed to manipulate the images, including changing the brightness, contrast, and zoom of the image. The gradings by experts were assumed correct and they were used as reference. Each of the AI-providers had defined their own cutoffs, which were used in the analysis. The AI-based result alternatives were non-referable, referable or ungradable. Some of the algorithms returned results per person instead of an eye. Therefore, all the AI-based results were analysed per person. With the algorithms returning eye-specific results, the more severe result was used in the comparison versus human grading.

For the assessment of AI algorithms, 24 providers with automated AI-based DR screening systems were offered the opportunity to participate in the study. The details of the study were provided in a letter sent to each provider and a more in-depth explanation of the comparison study was provided verbally. This included, for example, the setting of threshold for referable disease and human grading being the true value. Of the providers approached, 21 completed the study and several of them agreed to publish their names (AEYE Health, New York, NY; AIScreenings, Paris, France; Aurteen Inc., Alberta, Canada; iHealthScreen, Richmond Hill, NY; OphtAI, Paris, France; Ophthalytics, Atlanta, GA; Orbis International, New York, NY; REACH-DR, Philippines & Joslin Diabetes Center, Boston, MA; Retmarker SA – Meteda Group, Rome, Italy; Insight Eye, Somerset, NJ; Thirona Retina, Nijmegen, Netherlands; ULMA Medical Technologies, Oñati, Spain; Viderai, Ostrava, Czech Republic; VITO, Boeretang, Belgium). It was agreed before the study initiation, that the identity of each AI-provider was masked along with its submitted algorithms, and algorithms were labelled from A to U in random order. All 21 algorithms had been trained and validated for DR screening by the providers. Eight of the algorithms had also been certified with a CE-mark (class I or class II).

Each of the screening algorithms were compared independently against human graders as reference when analysing real-world retinal imaging data. Twenty-one companies provided algorithms that analysed all images without any pre- or post-processing, regardless of image quality. As previously described, the result alternatives were non-referable and referable. If algorithm was not able to analyse any of the images of an individual, then the result returned was ‘ungradable’. The sensitivity and specificity of each algorithm in grading non-referable or referable DR were compared with grading by two experienced ophthalmologists (gold standard). To evaluate the diagnostic accuracy of the algorithms, screening performance measures included agreement on non-referable/referable DR grading, sensitivity and specificity. An ungradable rate was also calculated if algorithm was not able to return a result for all the subjects.

Means with 95% confidence intervals are presented for sensitivities and specificities. Youden’s index was used to rank the algorithms. All analyses were calculated by SPSS for Windows (IBM Corp., Released 2021, IBM SPSS Statistics for Windows, Version 28.0, IBM Corp., Armonk, NY; license obtained from University of Oulu).

## Results

A total of 1248 fundus images of 312 eyes from 156 patients were included in the study. Most of the eyes, 183 out of 312 (58.7%), had no DR, whereas mild NPDR was noted in 21 (6.7%) based on ophthalmologists’ grading. Thus, non-referable DR was documented in 65.4% of all cases. Moderate NPDR was noted in 66 (21.2%) of the eyes, one (0.3%) of the eyes had severe NPDR, and 41 (13.1%) had PDR composing a group of 34.6% of eyes with referable DR.

**Figure 1.**
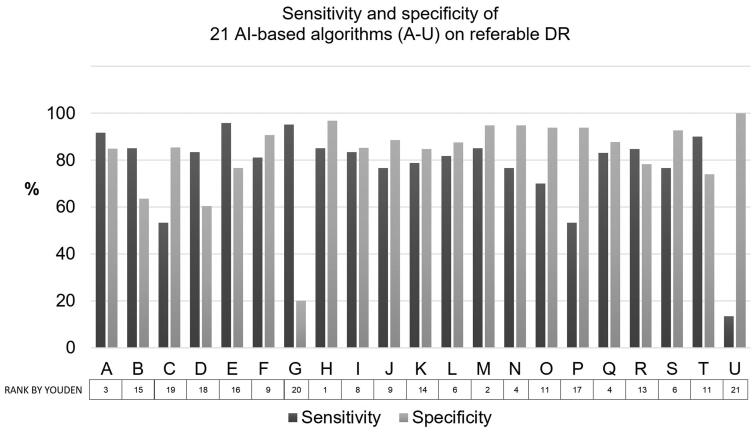
Sensitivity and specificity of 21 AI-based algorithms on referable DR. The algorithms were ranked by Youden’s index.

The 21 AI algorithms included in the study revealed the mean agreement of 79.4% in the classification of non-referable/referable DR (median 82.1%), but there was a wide variation between the lowest and highest values of the agreement; from 49.4% to 92.3%. The mean sensitivity of the algorithms was 77.5% (95% CI 69.1–85.8, range from 13.3% to 96.7%). The mean specificity was 80.6% (95% CI 72.1–89.2, range from 20.0% to 100.0%). Five of a total of 21 AI based algorithms, A, E, G, J and L, had ungradable images of rates 1.9%, 28.2%, 10.9%, 0.6% and 1.9%, respectively. Nineteen out of 21 (90.5%) AI algorithms resulted in grading for DR at least in 98% of the images. The sensitivity and specificity for each AI screening system are summarized in Figure 1.

When only the top five algorithms (A, H, M, N and Q) ranked by Youden’s index were analysed, the mean agreement on the non-referable/referable DR was 89.2% (median 89.3%), sensitivity 84.3% (median 85.0%) and specificity 91.3% (median 94.8%).

The retinal abnormalities other than DR, such as age-related macular degeneration, branch retinal vein occlusion, central retinal vein occlusion, were the most common causes for a false positive grading by AI. Moderate NPDR was the most typical cause for a false negative result from algorithms.

## Discussion

Recently, we have shown evidence of the feasibility of the handheld Optomed Aurora fundus camera in DR screening. According to current results, it seems that the camera is also suitable for AI-based automated DR screening. In a population of 156 subjects with diabetes, almost 60% of the participants had no DR and referable DR was noted in 35% of the patients. The sensitivity and specificity were 92% and 100%, respectively, in DR detection [18]. In agreement, other studies have suggested that easily movable handheld fundus cameras might serve as an alternative and cost-effective tool for organizing screening of DR especially in countries with low healthcare and resource levels [17,19,20]. The results of the current study are in line revealing good quality of the images and very low rate for the ungradable ones in a majority of the algorithms.

The results of the present study showed variability in the rates for sensitivity and specificity between 21 algorithms, but the mean values of 77.5% in sensitivity and 80.6% in specificity are reasonable. It is notable, though, that the best algorithms managed very well, while the poorest did not reach the acceptable level of performance in the current dataset. The sensitivity, specificity and rate for agreement on non-referable/referable DR all increased markedly when only the top five algorithms were measured suggesting that reliable algorithms of high quality exist despite the variability between the solutions. All the images captured with the handheld Optomed Aurora camera were totally unprocessed before the AI analysis, and the results might have been different if the dataset had been modified before the measurements. There is variation in the grading scales for DR severity used in the previous studies, which complicates the comparison of the results from the performance of AI algorithms [21,22]. For example, performance of IDx-DR differed significantly by using the grading scale according to EURODIAB resulting in 91% sensitivity and 84% specificity, whereas they were 68% and 86%, respectively, for ICDR [22]. This points out the importance of the grading guidelines since they significantly affect the outcome and performance of AI as well as the results published from various solutions. However, an adequate balance between high sensitivity and specificity is the key to establishing cost-effective screening programs. More cases of DR are missed if the sensitivity is low, and low specificity leads to a relatively large number of false positives demanding further examination, which consumes the resources that automated DR screening is trying to spare.

The primary starting point for implementation of automated screening systems into clinical use could be sorting out the fundus images with no DR or other pathologies from the ones with any DR. According to our results, this would at least halve the need for human grading and hence reduce the cost and time used for analysis since most of the patients, almost 60%, had no DR. Usage of AI systems have indeed been demonstrated to lower cost by at least partially replacing human graders, improving diagnostic accuracy and increasing patient access to DR screening [8,12]. Automated DR detection algorithms have several advantages over human-based screening; algorithms do not get tired and can grade thousands of fundus images a day. In addition, grading results are often provided within seconds to minutes of shooting the photographs. Nevertheless, human graders are still very likely needed to judge atypical or low-quality images and to ensure the quality of screening, and hence the completely automated DR screening may not actualize in clinical practice in the very near future.

There are several limitations of the study. Demographics of the participants were not collected and detailed clinical information of the study patients is lacking. Algorithms were evaluated anonymously which limits the detailed comparison of the properties of each algorithm. The accuracy of comparison might also be impacted due to limited knowledge about threshold used by AI to count something as referrable DR. Formal sample size calculations were not performed, which may be considered as a limitation. The number of patients included was estimated in a way that there were reasonable number of patients in each stage of DR. However, further studies of the AI based algorithms in DR screening in larger dataset are needed. The strength of the study is that performance of large number, 21, AI-based algorithms were compared. One may assume, whether the results and the order of the AI algorithms could be different if other cameras were used. In the current setting, however, the results obtained by Optomed Aurora are promising.

Our real-life results suggest that the performance of the algorithms may vary when measured against the selected testing dataset or unmodified, real-world data obtained from actual screening conditions. The limited performance of some of the algorithms in our study emphasizes the need for rigorous pre- and post-approval testing and external validation to sufficiently identify and understand the algorithms’ characteristics to determine suitability for clinical implementation. The knowledge and understanding of the possibilities and limitations of AI solutions is crucial for their successful use in a real-world setting: patient acceptability, data privacy, data protection, regulations, including medico-legal aspects, are among the issues that need to be considered [23]. Utilization of AI in ophthalmology is not limited to DR but may be applicable for earlier detection of age-related macular degeneration and glaucoma to improve the clinical outcomes of these common eye diseases.

## Conclusions

The performance of 21 AI algorithms varied considerably emphasizing the need for external validation of screening algorithms in real-world settings before their clinical application, although the best-performing algorithms could fulfil the requirements of DR screening recommendations. The implementation of AI is likely to improve the efficacy of DR screening.

## Data Availability

The data that support the findings of this study are available from the corresponding author, NH, upon reasonable request.
